# Roles of calpain-calpastatin system (CCS) in human T cell activation

**DOI:** 10.18632/oncotarget.13259

**Published:** 2016-11-09

**Authors:** Anna Mikosik, Aleksandra Jasiulewicz, Agnieszka Daca, Izabella Henc, Joanna E. Frąckowiak, Katarzyna Ruckemann-Dziurdzińska, Jerzy Foerster, Aurelie Le Page, Ewa Bryl, Tamas Fulop, Jacek M. Witkowski

**Affiliations:** ^1^ Department of Pathophysiology, Medical University of Gdańsk, Gdańsk, Poland; ^2^ Department of Pathology and Experimental Rheumatology, Medical University of Gdańsk, Gdańsk, Poland; ^3^ Department of Clinical and Social Gerontology, Medical University of Gdańsk, Gdańsk, Poland; ^4^ Research Center on Ageing, University of Sherbrooke, Sherbrooke, Quebec, Canada

**Keywords:** T cells, calpain, calpastatin, proliferation, cytokines, Immunology and Microbiology Section, Immune response, Immunity

## Abstract

The immune response is determined by the speed of the T cell reaction to antigens assured by a state of readiness for proliferation and cytokine secretion. Proliferation, apoptosis and motion of many cell types are controlled by cytoplasmic proteases - μ- and m-calpain - and their inhibitor calpastatin, together forming the “calpain-calpastatin system” (CCS), assumed to modify their targets only upon activation-dependent cytoplasmic Ca^2+^ increase. Contrastingly to this notion, using quantitative real time PCR and semiquantitative flow cytometry respectively, we show here that the CCS genes are *constitutively expressed*, and that both calpains are *constitutively active* in resting, circulating human CD4^+^ and CD8^+^ lymphocytes. Furthermore, we demonstrate that calpain inhibition in the resting T cells prevents them from proliferation *in vitro* and greatly reduces secretion of multiple cytokines. The mechanistic reason for these effects of calpain inhibition on T cell functions might be the demonstrated significant reduction of the expression of active (phosphorylated) upstream signalling molecules, including the phospholipase C gamma, p56Lck and NFκB, in the inhibitor-treated cells. Thus, we propose that the constitutive, self-regulatory calpain-calpastatin system activity in resting human T cells is a necessary, controlling element of their readiness for complex and effective response to antigenic challenge.

## INTRODUCTION

Permanent readiness of the resting, circulating T lymphocytes to mount an effective response against invading pathogens is an obvious evolutionary benefit. We show here that the *constitutive* activity of endogenous proteases - calpains - participates in and is necessary for keeping the T lymphocytes in the state of adequate alertness.

Two members of the calpain (calcium-dependent neutral cysteine protease) family named μ-calpain and m-calpain, are found in many mammalian tissues, including blood and immune cells [1, 2]. One of the most characteristic features of the activity of these two proteases is their absolute dependence (at least *in vitro*) on the availability of the required (micromolar and millimolar, respectively) concentrations of Ca^2+^ [1, 2]. an important notion here is that these enzymes, by limited proteolysis in single sites of the substrate peptides, modify these substrates and render them inactive or active rather than degrading them outright [1, 2, 3, 4]. adjustable, proteolytic the the control of the modification of transcription factors including NFκB, NFAT, c-fos, c-jun and STAT family [5-7]). indirectly, including, −2, BclxL, Bid, and This, in turn, leads to the observations that active calpains may participate in several pathologies, including the Alzheimer's disease, muscular dystrophies, cancers, and leukemias [9-13].

The putative involvement of μ- and m-calpains in “life-or-death” decisions and processes in many cell types is corroborated by the presence of a co-existing, specific, competitive cytoplasmic inhibitor - calpastatin [14]. It is of note that calpastatin in turn is specifically activated by active calpain through limited, Ca^2+^-dependent proteolysis [15, 16]. Calpains themselves are their own substrates in a self-limiting activation/degradation cycle [16, 17]. Thus, the whole system operates in a feedback loop. The balance must be strictly regulated, since the excess of any component can lead to cell malfunctioning and to the consequent progression towards diseases [12, 13, 16]. Together, the two calpains and calpastatin form a self-regulating, tightly controlled proteolytic unit called the calpain-calpastatin system (CCS).

Abnormal activity of calpains affects the migration and proliferation of cancer cells, as well as the intra-tumour angiogenesis and apoptosis [9, 10, 16, 18]. We have shown that increased amount and total activity of μ-calpain in the cells of chronic B-cell leukaemia (B-CLL) and childhood acute leukaemia blasts (ALL-B) prevent them from undergoing apoptosis and is conductive to their accumulation in a calpain inhibitor-reversible manner [12, 13]. Furthermore, the amounts of all three members of the CCS system in the peripheral blood T and B lymphocytes had been recently demonstrated to vary between young and elderly people as well as between the T cells of healthy adults and rheumatoid arthritis patients [15, 19]. We have suggested earlier that increased calpain activity might participate in the modified course of the cell cycle of the CD4^+^ T lymphocytes of elderly people [20]. Also other authors suggested a role for calpains' activity in the immune cells' functions, including adhesion, cytoskeleton rearrangement, activation and signal transduction [10, 18]. In fact, expression of calpains and calpastatin in human blood cells (including lymphocytes) is already known for almost three decades [21]. However, despite the relatively numerous already known facts regarding the CCS presence in human lymphocytes, calpains were so far never *directly* implicated in the control of the lymphocyte proliferation. Thus, in this work we not only demonstrate that CCS *is active in resting human peripheral blood T cells*, but also that this activity is essential for controlling their proliferation and multiple cytokine secretion, at least *in vitro*.

## RESULTS

### μ-calpain, m-calpain and calpastatin are present, their genes transcribed and both calpains are proteolytically active in unstimulated human peripheral blood T cells

**Figure 1:**
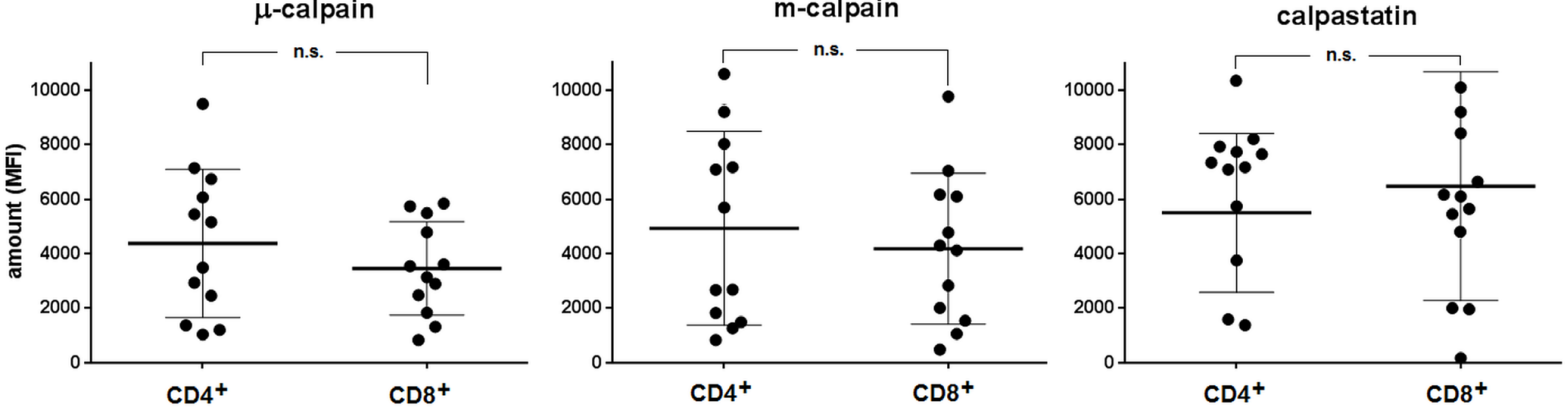
Similar relative amounts of μ- and m-calpain in resting CD4^+^ and CD8^+^ lymphocytes Calpain amounts were estimated by flow cytometry using appropriate anti-calpain and anti-calpastatin antibodies as well as appropriate surface staining as in Materials and Methods. CCS protein amounts are shown for each individual (•) and as means ^+^/− SD. Statistical significance of differences was assessed using unpaired T test. The differences were not statistically significant (n.s). N = 12.

As shown in the Figure 1, similar amounts of μ-calpain, m-calpain and calpastatin are present in the peripheral blood CD4^+^ and CD8^+^ T cells (Figure 1). Also, we did not observe any significant differences when the amounts of CCS proteins were compared among the CD4^+^ or CD8^+^ lymphocyte activation and differentiation states using the CD25, CD28, CD45RA, CD45RO, CD69 or CD95 expression (not shown). On the other hand, the amounts of μ-calpain in CD4^+^ and CD8^+^ lymphocytes significantly correlated (Pearson r = 0.720, *p* = 0.0083). Similarly significant correlations were found for the amounts of m-calpain (Pearson r = 0.894, *p* < 0.00001) and of calpastatin (r = 0.815, *p* = 0.001) in these two lymphocyte populations.

**Figure 2:**
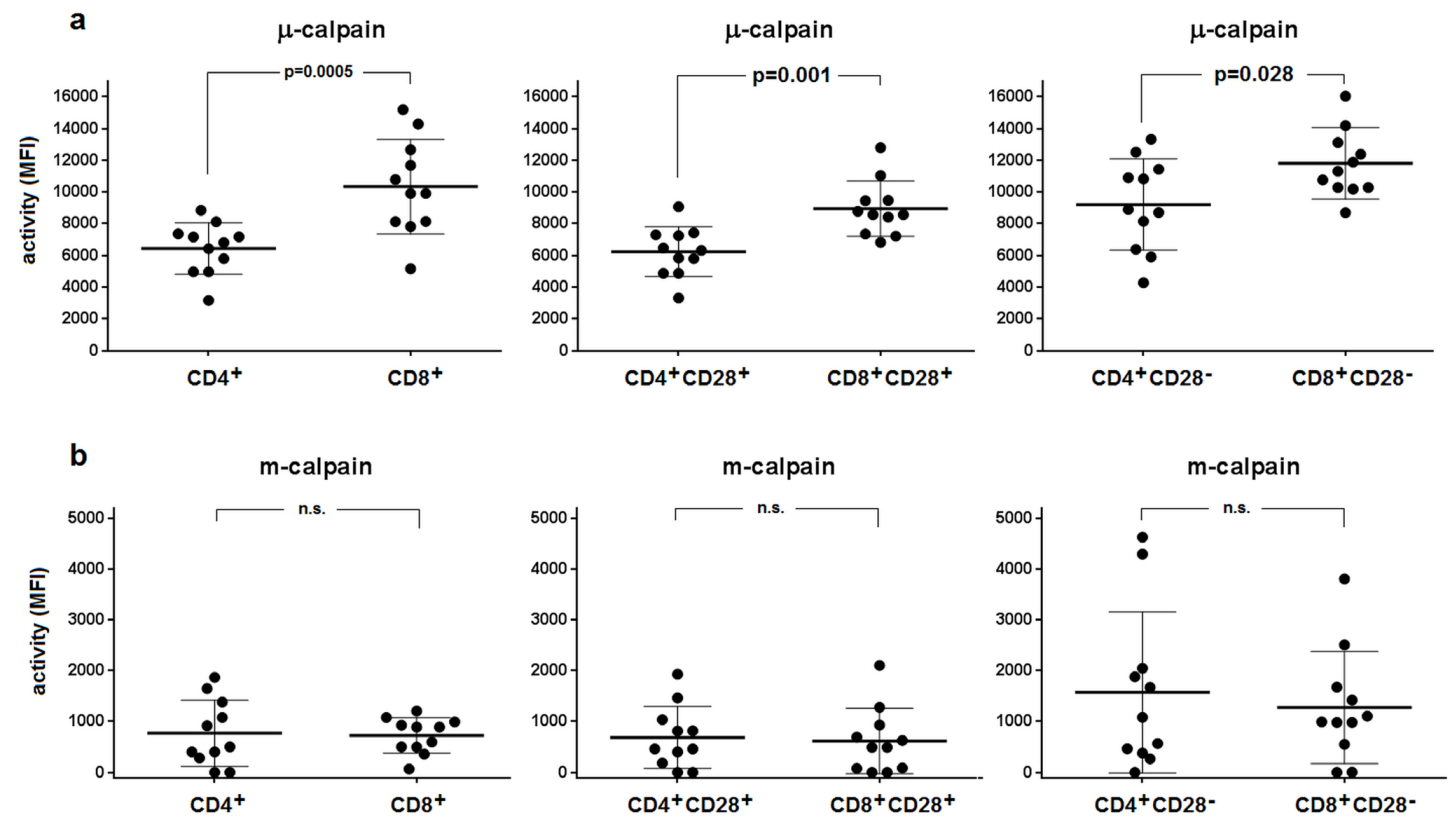
Relative activities of μ- and m-calpain differ between CD4^+^ and CD8^+^ lymphocytes and their CD28+ and CD28- subpopulations The calpain activities were measured cytometrically using CMAC-tBOC as a substrate and specific calpain inhibitors in the resting T cells defined by CD4, CD8 and CD28 expression, as described in Materials and Methods. **a.**- μ-calpain activities for CD4^+^ *vs*. CD8^+^ (left panel), CD4^+^CD28^+^ *vs*. CD8^+^CD28^+^ (middle panel) and for CD4^+^CD28^−^ *vs*. CD8^+^CD28- lymphocytes (right panel). The same order applied for m-calpain activities shown in **b.** Activities are shown for each individual (•) and as means ^+^/− SD. Statistical significance of differences was assessed using unpaired T test and the resulting p values for significant differences are shown. N.s. - not significant. N = 11.

Using the flow cytometry approach and CMAC-tBOC as a fluorogenic substrate detecting the activity of both calpains, we then attempted to assess the activities of μ- and m-calpain in the resting CD4^+^ and CD8^+^ T cells and in their subpopulations differing in the expression of CD28 (earlier shown to affect proliferative dynamics of CD4^+^ T cells [20]). We were able to demonstrate the μ- and m-calpain activities in all T cell populations tested (Figure 2). M-calpain activity was very significantly (*p* < 0.0001 for every pair tested) lower than that of μ-calpain in each T cell population studied (compare Figure 2a and Figure 2b). The resting activity of μ-calpain was significantly higher in CD8^+^ cells and in their CD28^+^ and CD28^−^ subpopulations than in the CD4^+^ lymphocytes and their respective subpopulations differing in CD28 expression (Figure 2a). It was also significantly higher in CD4^+^CD28^−^ than in CD4^+^CD28^+^ T cells (paired T test, *p* = 0.0027) as well as in CD8^+^CD28^−^ than in CD8^+^CD28^+^ T cells (paired T test, *p* = 0.0001). In contrast, the activities of m-calpain did not differ between resting CD4^+^ and CD8^+^ cells or between their respective CD28+ and CD28- subpopulations (Figure 2b). M-calpain activity was significantly higher in the CD8^+^CD28^−^ than in CD8^+^CD28^+^ T cells (paired T test, *p* = 0.003), but not when it was compared between CD4^+^CD28^+^ and CD4^+^CD28^−^ lymphocytes.

Activities of μ-calpain in CD4^+^ and CD8^+^ lymphocytes correlated significantly (Spearman r = 0.560, *p* = 0.038), as did its activity in the CD4^+^CD28^+^ and CD8^+^CD28^+^ T cells (r = 0.591, *p* = 0.028). Regarding m-calpain activities, significant correlation could be found only when these activities were compared between CD4^+^CD28^+^ and CD8^+^CD28^+^ cells (r = 0.753, *p* = 0.0075), but not for the total CD4^+^ and CD8^+^ populations. Correlations between μ-calpain and m-calpain activities in CD4^+^CD28^−^ and CD8^+^CD28^−^ lymphocytes did not reach statistical significance. Characteristically, the measured calpain activities did not correlate with the detected amounts of the CCS proteins (not shown).

**Figure 3:**
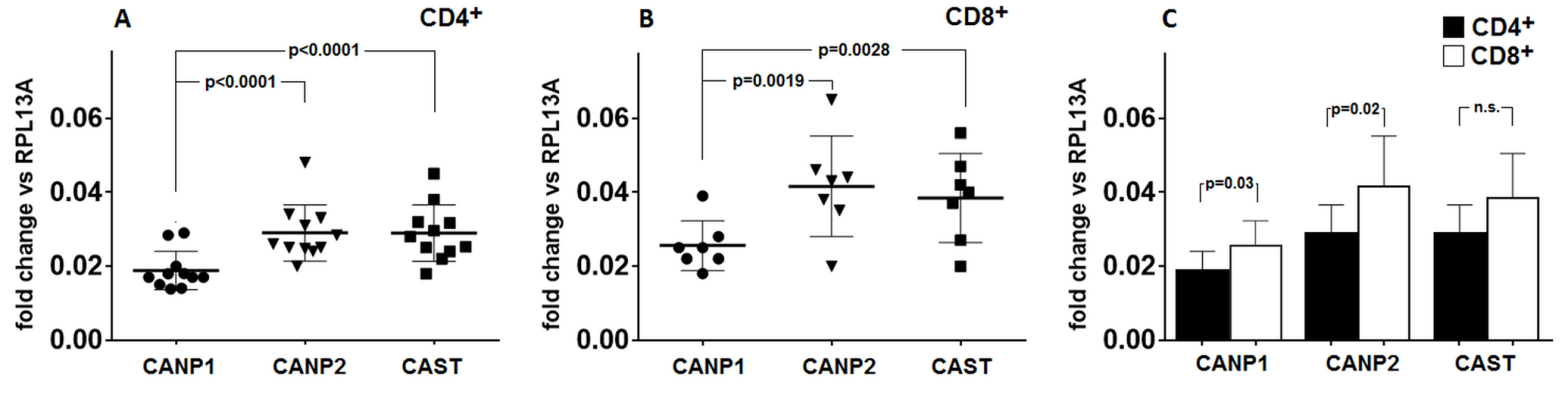
Level of transcription of CANP1 (μ-calpain) gene in resting CD4^+^ and CD8^+^ lymphocytes is significantly lower than these of CANP2 (m-calpain) and CAST (calpastatin) genes Resting CD4^+^ **a.** and CD8^+^ lymphocytes **b.** were purified from PBMC by FACS sorting and transcriptional activities of *CANP1* (•), *CANP2* (▼) and *CAST* (■) genes were quantified against that of constitutively expressed RPL13A gene by real-time PCR as described in Materials and Methods. Results of individual determinations as well as means ^+^/− SD are shown. Levels of *CANP1, CANP2* and *CAST* expression in CD8^+^ lymphocytes are higher than in CD4^+^ cells **c.** Wherever the difference was statistically significant (by paired T test), the p values are given in the Figure.

Based on the results of quantitative real-time PCR experiments, we have established that transcription of μ-calpain (*CANP1*), m-calpain (*CANP2*) and calpastatin (*CAST*) genes is *constitutive* in both the resting CD4^+^ and CD8^+^ cells (Figure 3a, 3b). Surprisingly, in both lymphocyte populations the transcription levels for CANP2 and CAST genes were significantly higher than that of CANP1 gene (Figure 3a, 3b). Transcription of *CANP1* and *CANP2*, but not that of *CAST*, was significantly higher in resting CD8^+^ than in resting CD4^+^ cells (Figure 3c). We did not find any significant correlation between the expression levels of *CANP1, CANP2* and *CAST* genes and amount or activity of any of the CCS proteins (not shown).

**Figure 4:**
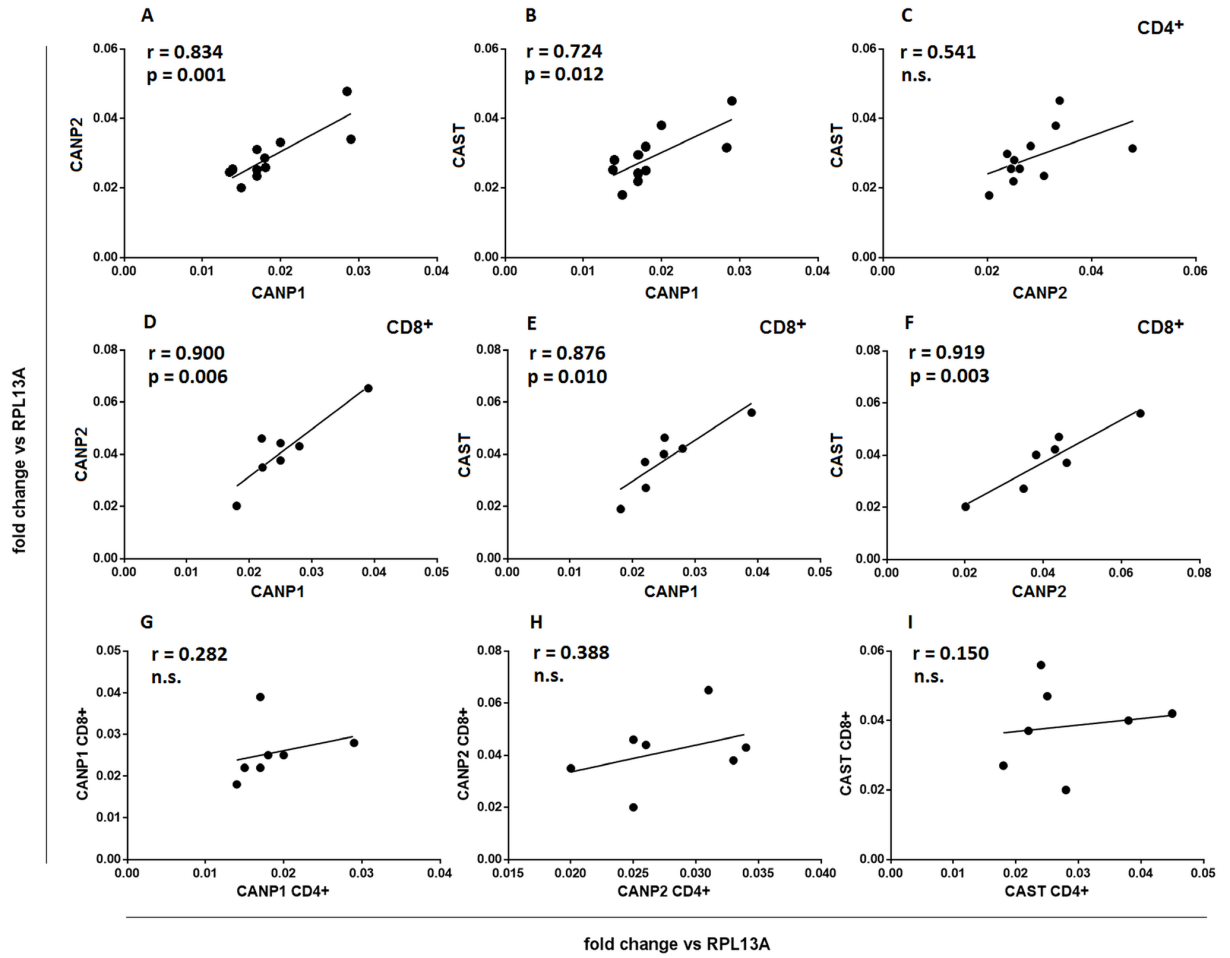
Expression of CCS component genes strongly correlates within, but not between CD4^+^ and CD8^+^ lymphocyte populations Resting CD4^+^ **a.**-**c.** and CD8^+^ lymphocytes **d.**-**f.** were purified from PBMC by FACS sorting and transcriptional activities of *CANP1*, *CANP2* and *CAST* genes were quantified against that of constitutively expressed RPL13A gene by real-time PCR as described in Materials and Methods. Each point (•) illustrates coordinated expression of two genes of interest for a single sample. **g.**-**i.** - same data as in **a.**-**f.**, reorganized to show lack of significant correlation between expression of CCS component genes in CD4^+^ and CD8^+^ cells of an individual. Pearson's r, regression curves and p values for significant correlations are shown. N.s.- not significant.

We further investigated whether the expression of the different CCS component genes is correlated within the cells belonging to either CD4^+^ or CD8^+^ population as well as between these populations. Data presented in the Figure 4a-4f show that there is a strong positive correlation between the expression of *CANP1, CANP2* and *CAST* within both the resting CD4^+^ and CD8^+^ cells, but there is no such correlation when the expression of each gene is compared for CD4^+^ and CD8^+^ cells of each study subject (Figure 4g-4i).

### Inhibition of calpain activity significantly and differently reduces proliferative efficiency of peripheral CD4^+^ and CD8^+^ T lymphocytes

**Figure 5:**
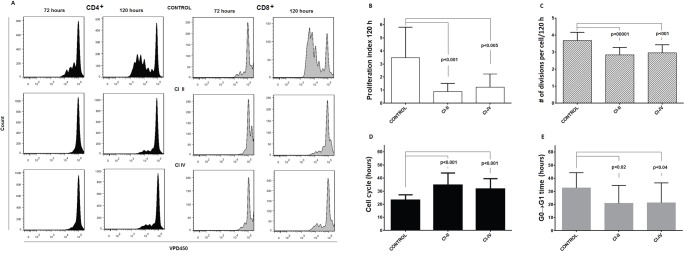
Calpain inhibition decreases proliferation of peripheral blood T cells *in vitro* **a.**- representative histograms of VPD450 fluorescence of gated CD4^+^ (black histograms, left panels) and CD8^+^ lymphocytes (grey histograms, right panels) obtained after 72 and 120 hours in culture where PBMC were stimulated with immobilized anti-CD3 and untreated or treated with 10 μM calpain inhibitor II (CI II) or with 10 μM calpain inhibitor IV (CI IV) as described in Materials and Methods. **b.**-**e.** significantly reduced dynamic proliferation parameters of CD4^+^ cells treated with calpain inhibitors as above. **b.** - reduction of proliferation index after 120 hours in culture (PI 120), **c.** - reduction of average number of divisions performed by a dividing precursor cell during 120 hours of culture, **d.** - increased average length of a cell cycle, **e.** - decreased average time from contact with stimulant to the onset of G1 phase of first division. Means ^+^/− SD are shown, N = 12. P values obtained using the paired Student T test are shown for significant differences.

When PBMC were stimulated with immobilized anti-CD3 antibody and concomitantly either μ- and m-calpain or only m-calpain activities were inhibited, the ability of CD4^+^ and CD8^+^ cells to proliferate had been significantly reduced. Figure 5a shows representative histograms of proliferation-dependent changes of VPD450 fluorescence in gated CD4^+^ or CD8^+^ lymphocytes from PBMC cultures performed without or with calpain inhibitors. These results clearly demonstrate that the effect of calpain inhibition manifested by the reduction of the number of divided generations as well as by the reduction of the number of divided cells in each generation is already evident after 72 hours in culture and even stronger after 120 hours. Analysis of the dynamic parameters of proliferation [22] of CD4^+^ and CD8^+^ lymphocytes in these cultures demonstrated that the proliferation index at 120 hours in culture (PI 120), corresponding directly to the number of cells “produced” by dividing responder cells (precursors), is significantly (on average more than threefold) reduced in cultures exposed to either calpain inhibitor CI-II or CI-IV (Figure 5b). Reduction of cell yield by calpain inhibitor treatment may in part be explained by the significant reduction of the number of divisions performed by an average T cell (Figure 5c), as well as directly associated with elongation of mitotic cycles (Figure 5d). Finally, a bit surprisingly, the preparatory stage prior to the onset of the first mitotic cycle (so called G0→G1 transition time) was significantly shorter in inhibitor-treated lymphocytes (Figure 5e). There was no significant difference in the effects of CI-II and of CI-IV on any of the proliferation parameters studied (Figure 5b-5e). We did not find any significant correlation between resting μ- or m-calpain activities and proliferation parameters (not shown).

### The effect of calpain inhibition on T cell proliferation does not depend on increased apoptosis of these cells

**Figure 6:**
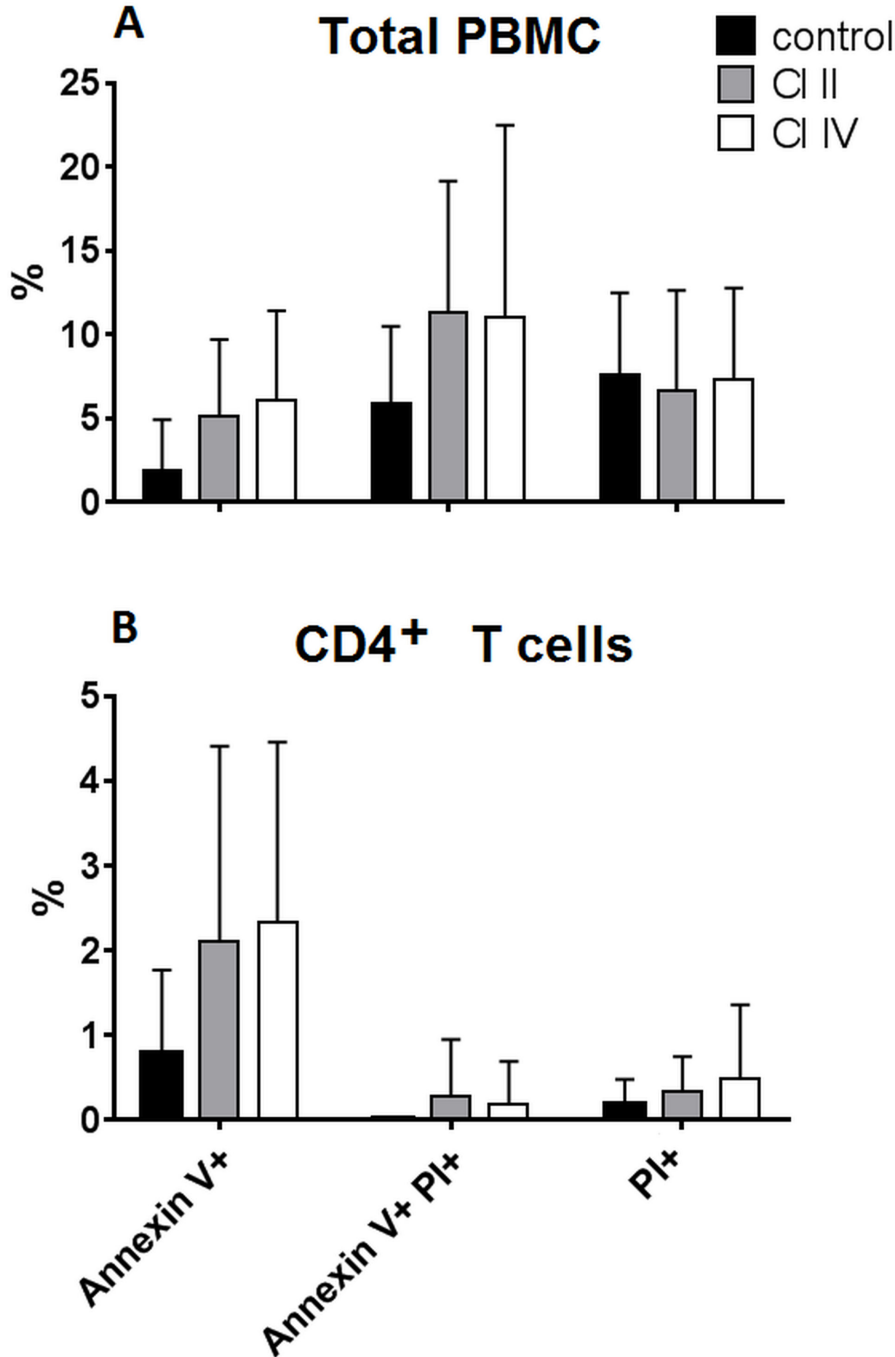
Calpain inhibitor treatment does not increase significantly the mortality of lymphocytes cultured *in vitro* PBMC were stimulated *in vitro* for 120 hours with immobilized anti-CD3 antibody and untreated (control), or treated with either 10 μM calpain inhibitor II (CI II), or with 10 μM calpain inhibitor IV (CI IV) as described in Materials and Methods. Bars reflect mean percentages of Annexin V- positive (early apoptotic), Annexin V and propidium iodide (PI)-positive (late apoptotic) and PI-positive (necrotic) cells among PBMC **a.** and among CD4^+^ T cells **b.** Standard deviations are marked. N = 12. The differences were not statistically significant.

We have shown before that inhibition of calpains in chronic and acute lymphoid leukemias induced significant apoptosis of the leukemic blasts [12, 13]. Thus, the observed anti-proliferative effect of calpain inhibitor treatment on normal T cells could conceivably be the induction of massive apoptosis, similar to observed in leukemic lymphocytes. The calpain inhibitors could also be simply toxic to the lymphocytes at the concentration used. Therefore we have analyzed the proportions of early apoptotic, late apoptotic and necrotic cells (distinguished by Annexin-V and propidium iodide staining) among the PBMCs and gated CD4^+^ lymphocytes after 120 hours of anti-CD3 stimulation with or without calpain inhibitors. The results shown in the Figure 6 demonstrate that treatment of PBMC with 10 μM CI II or with 10 μM CI IV did not significantly increase the proportions of dying cells among total PBMC lymphocytes (on average about 10-12%) as well as among gated CD4^+^ T cells, which rarely exceeded 4% for early apoptotic cells and were always below 2% of late apoptotic and necrotic cells. CI treatment did not induce apoptosis in the CD8^+^ cells either (not shown). Thus, we conclude that increased mortality of calpain inhibitor-treated lymphocytes is not the reason behind their significantly decreased proliferation.

### Inhibition of μ-calpain, but not of m-calpain, significantly reduces the secretion of multiple cytokines

**Figure 7:**
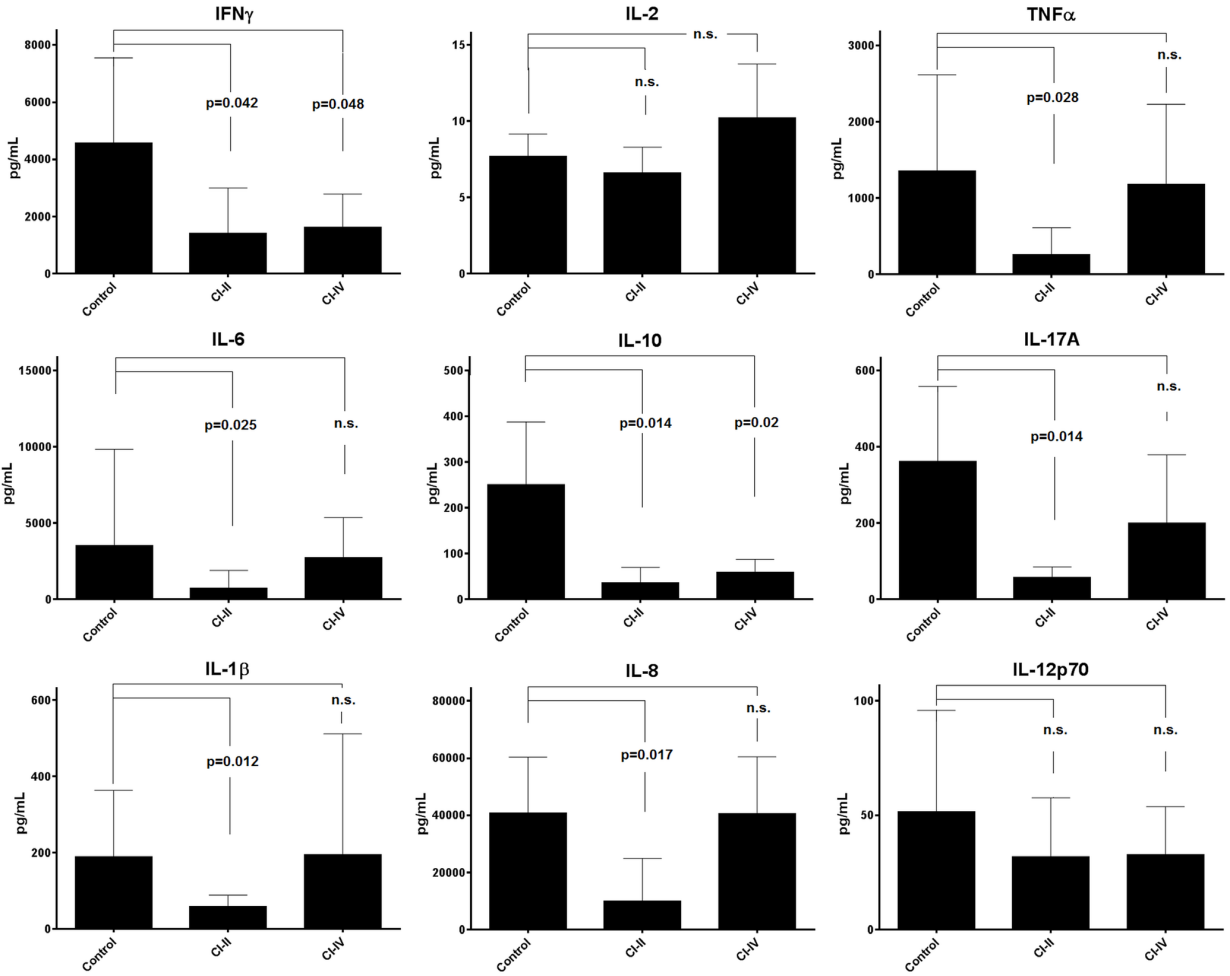
Calpain inhibitors exerts differentiated effect on cytokine production by stimulated PBMC Concentrations of various Th1, Th2, Th17 and proinflammatory cytokines (indicated in respective panels) were estimated using the CBA Flex™ cytometric technique as described in the Materials and Methods. Mean concentrations (bars) and standard deviations (whiskers) are indicated. P values are given in the panels whenever the difference between control and CI-treated cytokine concentration was statistically significant. N.s. - not significant. Student T test with Welch's correction was applied. N = 11 for each cytokine determination.

We have checked next if calpain inhibition may affect the ability of stimulated T cells to manufacture cytokines. Th1 (IFNγ, IL-2, TNFα), Th2 (IL-6 and IL-10), and Th17 (IL-17A) were analyzed as representative for main functional classes of the Th cells. In addition, monocyte/macrophage-derived cytokines: IL-1β, IL-8 and IL-12p70 were also assessed. Interestingly, the reaction of the production of different cytokines to calpain inhibitor treatment was diverse. Thus, treatment with CI-II resulted in significant reduction of the concentrations of most cytokines studied, with the notable exception of IL-2 and IL-12p70 (Figure 7). On the other hand, presence of 10 μM CI-IV in the PBMC cultures resulted in significantly reduced production only in the case of IFNγ and IL-10 (Figure 7). The levels of IL-6 and IL-10 secreted over 120 hours in PBMC cultures that were not treated with calpain inhibitors correlated negatively with resting μ-calpain activity in the CD4^+^ cells (Pearson's r = −0.764, *p* = 0.01 and r = −0.721, *p* = 0.019 respectively). Activity of μ-calpain in these cells correlated also with the concentration of secreted IL-2 (r = 0.591); however, this value had not reached statistical significance. Surprisingly enough, the resting activity of m-calpain in the CD4^+^ lymphocytes strongly positively correlated with the amount of secreted IFNγ (r = 0.953, *p* = 0.012). These correlations were not seen for the CD8^+^ lymphocytes. One has to stress here that the levels of IL-2 and IL-12p70 in PBMC cultures underwent only a small, not significant reduction in response to CI-II treatment.

### Inhibition of μ- or m-calpain significantly reduces the phosphorylation of signalling molecules in resting T cells

**Figure 8:**
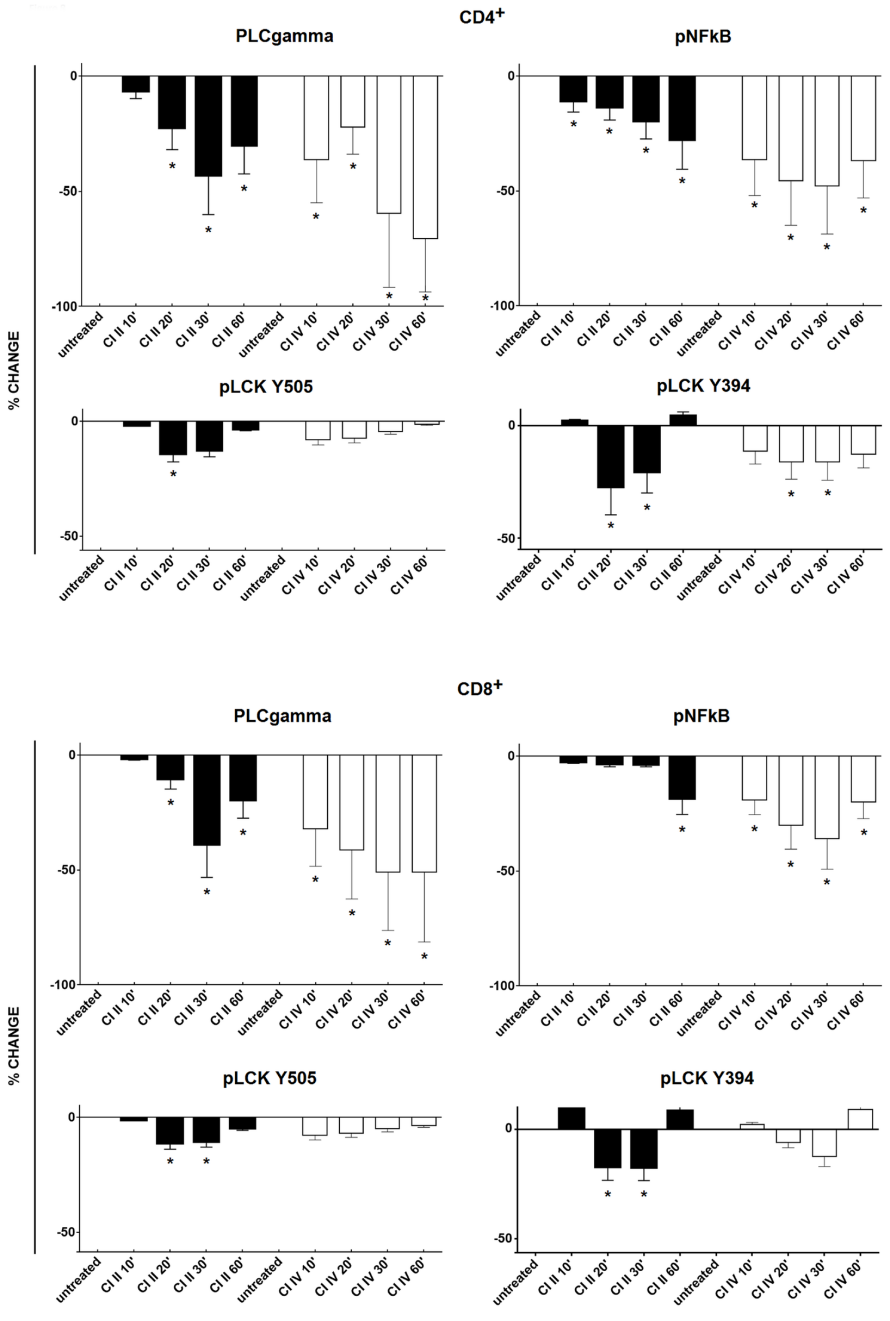
Calpain inhibition reduces the phosphorylation of key signaling molecules in resting peripheral T cells Resting PBMC were treated with either 10 μM calpain inhibitor II (CI II), or with 10 μM calpain inhibitor IV (CI IV) for 10, 20, 30 and 60 minutes or left untreated and then processed for cytometric estimation of the expression of phospho-PLCgamma (pPLCgamma), phospho-NFκB (pNFκB) or phospho-p56Lck (pLck; phosphorylation detected at Y505 and Y394 residues). Raw data were converted to percent deviation from the values obtained for untreated cells, expressed as % change (negative values indicating decreased expression) and shown as black bars for CI II and as white bars for CI IV, +/− SD. Upper panels illustrate changes in CD4^+^ and lower panels in CD8^+^ lymphocytes. Statistical significance of differences between untreated and calpain inhibitor (CI)-treated cells was estimated using Kolmogorov-Smirnov test as described in the Materials and Methods. Asterisks * correspond to *p* < 0.05.

In order to describe the molecular mechanisms behind the effects of calpain inhibition in resting T cells described above, we have investigated whether the inhibition of calpains, sufficient to curb T cell proliferation and cytokine secretion, does affect the activation (phosphorylation) of some of the signalling molecules. We studied the most significant signalling molecules as the p56Lck being the first tyrosine kinase in the TCR/CD3 pathway and the PLCγ responsible for the Ca^+^ mobilization, and finally the NFκB, a transcription factor common to the TCR and CD28 pathways. We have found that the phosphorylation status of PLCγ, NFκB and p56Lck, indicating their activation status in the resting T cells, is significantly reduced in resting CD4^+^ and CD8^+^ lymphocytes treated by calpain inhibitors (Figure 8). The level of inhibition was dependent on time of T cell contact with the inhibitors and the strongest was observed between 20 and 30 minutes of treatment. Inhibition of PLCγ and NFκB phosphorylation were apparently greater than that of p56Lck. The effects of calpain inhibitors CI II and CI IV on the phosphorylation of either signalling molecule did not differ significantly and neither did the effects exerted upon CD4^+^ and CD8^+^ lymphocytes. Interestingly, the reduction of the resting activation of p56Lck by calpain inhibition was independent on the phosphorylation site of Lck (Y394: activation or Y505: inhibition).

## DISCUSSION

Ability of the resting immunocytes to constantly maintain maximal functional readiness is clearly an evolutionary benefit. Circulating T cells, while remaining in the quiescent G_0_ stage, are ready to build the effective proliferative and secretory activities within hours from an antigenic challenge. Thus, it is conceivable that an intracellular system maintaining this readiness is operating in the resting, patrolling T cells. The calpain-calpastatin system (CCS) has long been recognized as participating in multiple intracellular processes, including apoptosis, movement, and proliferation (reviewed in [1, 16]), which makes it an interesting subject for studies of the immune system readiness. μ- and m-calpain were first detected in human mononuclear blood cells about three decades ago [21], and later demonstrated in T cell-derived cell lines [23], MHC-restricted T cell clones [24] and T cells infected with the HTLV-1 virus [11]. We have shown strong anti-apoptotic role of excessively expressed μ-calpain in human chronic and acute leukemias derived from the lymphoid (B-cell) lineage [12, 13], as well as the expression and the age-dependent changes in the amounts of CCS proteins in human peripheral blood lymphocytes [19] and, in a preliminary report, increased activity of calpains in the T lymphocytes of rheumatoid arthritis (RA) patients [15]. We have also hinted at possible role of the changed calpain activity in characteristically modified proliferative dynamics of CD4^+^ T cells of aged people [19, 20]. Calpain function in activated immune cells was studied also in multiple sclerosis and correlations with proinflammatory events in these patients were found [25]. Other authors had since described the role of calpain activity in the T cell cytoskeleton maintenance, cellular movements and related LFA-1 signalling in stimulated lymphocytes [18]. So far however, despite decades of relatively random studies, no conclusive report on the role of calpain activity in the human T cell proliferation and cytokine secretion is available and the published data remain controversial.

The expression of CCS genes and their products in human lymphocytes is still considered as negligible; as late as in 2009 Noma et al. had reported that calpain was constitutively present and active only in human monocytes, but not in lymphocytes [26]. Another recent study had concluded that “the conventional μ-and m-calpains are also expressed although at lower or almost undetectable amounts” [27]. In contrast, we have demonstrated here for the first time that not only *substantial amounts* of CCS proteins can be detected in each subpopulation of resting, unstimulated peripheral blood CD4^+^ and CD8^+^ T cells, but these cells also exhibit *constitutive activity* of μ-calpain and m-calpain. The *CANP1, CANP2* and *CAST* genes are *constitutively* transcribed in peripheral blood T lymphocytes, possibly to sustain the continuous production of new calpain molecules, known to self-degrade on activation [16, 17]. We were also able to show that the expression of these genes is significantly correlated within either lymphocyte population. Human *CANP1, CANP2* and *CAST* genes are located on 11^th^, 1^st^, and 5^th^ chromosome respectively [1], so their apparent co-expression cannot be related to a positional effect. Furthermore, it was reported that all of them contain the GC-rich sequences (GC boxes), which characterize other constitutively expressed genes [1]. It was also shown that the promoter regions of all three genes contain domains able to bind common transcription factors, including Sp-1, AP-1, NRF-1, GATA-1, and NFκB, which themselves are constitutively expressed in the T cells [1, 28, 29]. Taken together, these facts might be responsible for the *constitutive* expression of CCS genes in resting human T cells.

A canonical thinking about the activities of μ- and m-calpain is that they require micro- and millimolar intracellular concentrations of Ca^2+^ for activation. On the other hand, typical average concentration of Ca^2+^ in the cytoplasm of a lymphocyte is around 100 nM, i.e., at least an order of magnitude or more lower than that ostensibly required for activation of calpains. This consideration probably explains why the papers describing calpain activities in the lymphocytes concentrated on mitogen- or even calcium ionophore-stimulated cells, where the ensuing “calcium signals” may approach micromolar concentrations [24, 30-33]. How then activity of not only μ-calpain, but - as we show here - also m-calpain is at all possible in the *resting* T cells? It was elegantly demonstrated recently that each single ligation of TCR/CD3 complex by a cognate MHC-presented epitope, while not sufficient to invoke clonal expansion and cytokine secretion, leads to activation of protein kinases, as well as hydrolysis of inositol phosphates releasing the IP3. In turn, IP3 directly affects the ER calcium channels operating the store-operated calcium entry (SOCE), which results in the release of minute but measurable amounts of Ca^2+^ [32, 33]. This creates transient cytoplasmic microdomains, where there might be enough Ca^2+^ for calpain activation. The mechanism involves activation of the recently described ORAI1 channels, which depends on their interaction with the stromal interaction molecules (STIM), located in the ER membrane [34-36]. It was reported that mutations of either ORAI1 or STIM genes lead to channelopathy-dependent immunodeficiency [34], which conceivably could be at least in part due to reduced T cell proliferation and cytokine secretion depending on decreased calpain activation. Additionally, modulation of the intracellular Ca^2+^ oscillation at rest by K^+^ channels might also increase it to the level when calpains may be activated [37]. Calpains seem also to require contact with membrane lipids for full activation, but binding of even minute amounts of Ca^2+^ to the membrane phospholipids changes their properties and facilitates activation of membrane-bound calpain by reducing its need for Ca^2+^ [33, 38]. It was also suggested that m-calpain can actually be activated by active μ-calpain [4]. A corroborative evidence for direct relation between the activities of calpains and of ORAI1 channels had been demonstrated in murine model of Duchenne muscular dystrophy, where inhibition of ORAI1 by its specific inhibitor BTP-2 greatly reduced the cytosolic μ-calpain activity in the muscle fibres [39], and in skin keratinocytes, for which Ca^2+^- and calpain-dependent pathway is necessary for proliferation, differentiation and migration [40].

*Resting* cytoplasmic levels of Ca^2+^ had not been reported to significantly differ in human CD4^+^ or CD8^+^ cells or in their subpopulations. We were also unable to find convincing published evidence towards different levels of stimulation-elicited calcium signals between human peripheral blood CD4^+^ and CD8^+^ lymphocytes. In contrast, it is well known that *stimulated* memory T cells exhibit lower levels of Ca^2+^ signals than naïve T lymphocytes; however, there is no such difference between *resting* naïve and memory cells [41]. Previously, we did not see differences in the amount of CCS proteins between naïve and memory CD4^+^ lymphocytes [19]. Consequently, we had not pursued to study here the relation of calpain activity to the naïve and memory T cell phenotypes.

On the other hand, as mentioned above, in the stimulated T cells the average concentration of ionized calcium approaches micromolar [24, 30-33]. These activation-dependent calcium signals most certainly activate calpain above its resting activity state which we describe here. However, calcium signals only last a few minutes directly after TCR/CD3 triggering, during this time participating in the cascade of intracellular events leading to the formation of signalosomes, which culminates in the activation of transcription factors. Based on our experiments, already 1 hour after contact with the mitogen the T cell calpain activities return to these seen in the resting cells prior to stimulation (not shown). The mechanism would be the known autolysis of activated calpains on one side and constitutive transcription of the *CANP* genes resupplying the cells in new calpain molecules on the other. This once again supports our notion that high (calcium signal-dependent) calpain activity is necessary only for certain processes in an activated T cell (like fast activation of these transcription factors and stimulating migration), while lower, but constitutive calpain activity primes these cells facilitating their response.

It is possible that mostly μ-calpain is active in the resting T cells, while m-calpain is more significantly activated by calcium signal only. This would explain the effectiveness of CI IV (m-calpain inhibitor) in preventing proliferation of the mitogen-stimulated T cells (Figure 5).

Our results indicate that inhibition of *constitutive* activity of either calpain greatly reduces the numbers of proliferating T cells and the number of divisions they make in response to polyclonal stimulation. It must be stressed that these effects of constitutive calpain activity are not a simple consequence of protecting the T cells from activation-induced apoptosis, as calpain inhibition did not significantly increase apoptosis of stimulated T cells.

As we show here, μ-calpain (but not m-calpain) inhibition significantly reduced the production of certain cytokines by stimulated T cells. The list of affected cytokines includes those involved in inflammatory cells' stimulation (Th1, and Th-17, mainly IFNγ, TNFα and IL-17A respectively), but also some Th2 ones (IL-6, IL-10). According to a recent paper, over-expression of calpastatin suppresses the production of IL-6 and the development of Th17 T cells in mice, by reducing the NFκB and increasing STAT5 signalling [42]. Downregulation of Th1/Th17 cytokines by calpain inhibition leading to downregulation of IDO gene was also seen in the PBMCs of human multiple sclerosis patients [25].

The selective effect of calpain inhibition on cytokine secretion by T cells notably does not involve IL-2 (which eliminates one simple, mechanistic explanation of reduced proliferation of calpain inhibitor-treated T cells) and IL-12p70. Decrease of both IL-2 and CD25 mRNA production and of IL-2 secretion by calpain inhibition in activated human PBMC was reported in one early paper [30]. However, these authors used calpeptin rather than the tripeptide calpain inhibitors used here. It was recently reported that calpeptin directly (not *via* interference with calpain activity) inhibits the mitochondrial Ca^2+^-activated K^+^ channel (BK), leading to inhibited mitochondrial respiration and impaired cellular energy production [43].

The inhibitory effect of calpain inhibition on the production of proinflammatory cytokines may suggest an involvement of monocyte/macrophages, in which we did also see activities of μ- and m-calpain (not shown). However, reduced secretion of IL-1β by monocytes may be the consequence of the greatly reduced secretion of IL-1β-stimulating IL-17A and IFNγ [44] by CI-treated lymphocytes.

The functional importance of the constitutive calpain activity in resting T cells seems to be corroborated by the fact that it is tightly controlled by calpastatin, first activated to perform as calpain inhibitor and then degraded by calpain in the process [15, 16]. The *CAST* gene is constitutively transcribed in T cells and the cumulative amount of detectable calpastatin is stoichiometrically relevant. Modified μ-calpain-calpastatin balance in the graft-infiltrating T cells has recently been demonstrated to participate in acute kidney allograft rejection [45]. We can speculate that the role for constitutive CCS genes' transcription would be to maintain constant levels of CCS proteins in the resting T cells, countering their constant depletion by constitutive activity. The goal of this permanent supplementation of the immune cells in CCS proteins would be to maintain *constant level of calpain activities* to keep the cells in the state of homeostatic readiness to respond to antigenic challenges.

How then the constitutive calpain activity in resting T cells could facilitate their proliferation and cytokine secretion in response to stimulatory signals, and be responsible for maintaining their readiness for response to antigens? It has been reported that calpain itself degrades IκB, rendering NFκB more active [5]; also, it regulates STAT signalling [7]. Calpain inhibition was reported to activate MAP kinases: Erk, p38, JNK, PI3K/Akt and others, but only in monocytes and not in lymphocytes [26]. Still, knowing that the kinases listed above are crucial for appropriate T cell activation [26, 46], it is conceivable that they would also be affected by calpain activity in these cells upon stimulation. On the other hand, proteolytic modulation of the same protein may result in its activation or inhibition depending of the extent and duration of calpain activity [16, 24, 47]. Therefore it is conceivable that the abovementioned kinases are in fact also activated by calpain activity in the resting T cells, and inactivated by transiently high calpain activity in the recently activated lymphocytes, possibly in order to limit the strength of the intracellular signals. This is the case for protein kinases C (PKC), where proteolytic modulation by calpains has a hormetic effect: low activity renders more PKC molecules active, while high activity of the protease degrades them [26, 47].

The kinases and transcription factors mentioned above are relatively downstream along the signalling pathway from the TCR to the T cell nucleus, and therefore less likely to be the elements of cytoplasmic machinery maintaining the T cell readiness at the resting state. Therefore, we have tested if calpain inhibition in unstimulated T cells would affect the activation status of two molecules upstream in this signalling pathway: tyrosine-phosphorylated forms of phospholipase C gamma (pPLCγ) and p56Lck, in addition to the phosphorylated NFκB. We have demonstrated that calpain inhibition reduces significantly and in a time-dependent manner the phosphorylation of all three of these molecules in both resting CD4^+^ and CD8^+^ lymphocytes (Figure 8). Thus, by inversion, we can say that resting calpain activity is necessary for maintenance of adequate resting activation levels by maintaining tyrosine phosphorylation of PLCγ, NFκB and Lck, which ascertains sufficient level of readiness of the T cell signal transduction mechanisms. In fact, our data are supported by earlier report stating that the p56Lck molecule is already autophosphorylated at Y394 (considered an activation-associated site) in resting T cells [48]. Calpain activity would, therefore, be necessarily affecting these phosphorylation events, possibly by modifying the Lck molecule itself or even further upstream (at the CD3 molecule ITAM domains) [49]. Resting calpain activity might also be involved in reduction of the available activity of SHP-1 phosphatase, itself known to participate in the negative feedback involving the pLck phosphorylation at Y505 and Y394 and leading to decreased T cell activation in aging [50], and possibly other protein thyrosine phosphatases (PTPs) [51, 52].

The relatively widely, although controversially described effect of calpain activity in the lymphocytes concerns their migration. Thus, Svensson et al. demonstrated that the *m-calpain* activity is necessary for migration of activated T cells in an ORAI-1 channel-independent fashion [31]. On the other hand, a recent paper corroborates the notion of the potential role of ORAI-1 channel - dependent *μ-calpain* activity in the T cell migration [36]. However, both these datasets were generated only from activated, expanded T lymphoblasts, so it is an effect associated with high calpain activity elicited by calcium signals in proliferating cells [31, 36]. Still, these findings point at possible different roles of both calpains' activities in the T cells, corroborating our observations.

Summarizing, we suggest that the *constitutive* activity of calpains in resting T cells *primes them* for both proliferative and secretory function occurring during the actual T cell-dependent immune response, by maintaining them in constant alertness *via* modifying the signalling molecules involved in early signal transduction pathways. This may produce an evolutionary advantage over the cells where the relevant genes (and resulting functionalities) would have to be activated only upon strong antigenic signals. Thus, pharmacological modulation of resting calpain activities may represent a mean to influence T cell functions in physiological or pathological conditions.

## MATERIALS AND METHODS

### Material

Whole blood samples from 12 healthy, middle-aged individuals (6 F, 6 M; average age 47.2^+^/−7.7 years) were collected in EDTA-Vacutainer™ tubes. Peripheral blood mononuclear cells (PBMC) were isolated by density gradient centrifugation on Histopaque™ (Sigma), washed, counted and further processed. All participants were free from acute or chronic infectious, inflammatory, autoimmune, or allergic diseases, as well as from metabolic diseases and malignancies.

The study was approved by the Local Independent Committee for Ethics in Scientific Research at the Medical University of Gdansk. The patients gave written informed consent for participation in the study. The procedures were in accord with the *Helsinki Declaration of 1975*, as revised in 2008. All in accordance with

### Flow cytometry analysis of CCS protein amount

The analysis of relative amounts of the CCS proteins in various populations of resting peripheral blood lymphocytes was done as described before [19]. Briefly, in order to assess if the detailed T cell phenotype (especially different expression of CD28 or CD45RA [19]) would relate to CCS amount, the PBMC were surface-stained at 1 μg antibody/10^5^ cells for 30′ on ice in the dark with the following mouse anti-human monoclonal antibody-fluorochrome conjugates (all from Becton Dickinson Biosciences): CD3-APC-Cy™ 7 (clone SK7), CD4-PerCP (clone L200), CD8-V500 (clone RPA-T8), CD25-PE-Cy™7 (clone M-A251), CD28-APC (clone CD28,2), CD45RA-V500 (clone HI100), CD45RO-APC (clone UCHL1), CD69-PE-Cy™ 7, (clone FN50), and CD95-V450 (clone DX2) and with appropriate isotype controls. Stained cells were fixed/permeabilized with 2% paraformaldehyde and 0.25% saponin (Sigma Aldrich, Germany) in PBS. Intracellular labelling of CCS proteins was performed with mouse monoclonal anti-human μ-calpain (clone 15C10) or m-calpain (clone 28F3, both from Thermo Scientific) and anti-human calpastatin (clone CSL15, GeneTex, USA). All three antibodies were uniformly conjugated with Mix-n-Stain CF488A Antibody Labelling Kit (Biotium, Inc., USA) according to the manufacturer's instructions. Fluorescence of conjugates was detected as identical with that of fluorescein. Determination of the net values of the mean fluorescence intensities (MFIs) of the CCS protein-bound antibodies for semi-quantitative analysis and comparison was done using fluorescence-minus-one (FMO) controls (samples stained with all surface-staining antibodies, but not with anti-CCS ones). This technique allowed determining the ‘negative’ MFIs to be subtracted from the relevant ‘total’ MFIs of the samples stained with all antibodies including the anti-CCS, giving such “corrected” MFI values as a measure of relative amounts of CCS proteins.

### Calpain inhibition

Following membrane-permeant, tripeptide calpain inhibitors (CI) were used: calpain inhibitor II (CI II, N-Acetyl-Leu-Leu-Met-al; Calbiochem, UK) considered to inhibit both μ- and m-calpain with relative K_i_ equal to 120 nM and 230 nM respectively, and Z-Leu-Leu-Tyr-CH2F (Z = benzyloxycarbonyl), Calbiochem, UK. The concentrations of both CI were optimized at 10 μM, which gave maximal inhibition while not compromising the cell viabilities (See Results, section III below).

### Flow cytometry measurement of relative calpain activities

Fluorogenic substrate 7-Amino-4-Chloromethylcoumarin *t*-BOC-L-Leucyl-L-Methionine amide (CMAC-tBOC, Molecular Probes, USA [31] at 0.18 μM was used for measurements of relative calpain activity. One million PBMC were incubated in 1mLof complete RPMI culture medium for 1 hour at 37°C with or without one of the calpain inhibitors described above, then the CMAC-tBOC substrate was added and the cell suspension incubated for another 30′ at 37°C, washed twice with ice-cold PBS to stop the enzymatic reactions, stained with antibody conjugates (details above), washed and resuspended in ice-cold PBS. The cell suspensions were kept on ice until the moment of analysis and analyzed within 15′ from last wash. For quantification of relative calpains' activities in the T cells, CMAC-tBOC fluorescence (MFI) has been recorded as: 1 - fluorescence of cells incubated with no substrate (background), 2 - total fluorescence after incubation with CMAC-tBOC only, indicating all cytoplasmic activities capable of cleaving the substrate, 3 - fluorescence in the cells treated with CI II, and 4 - substrate fluorescence in the cells treated with CI IV. After subtracting background (1) fluorescence from all of the other ones (2-4), relative activity of μ-calpain was expressed as corrected MFI units obtained by subtraction of 3 from 4 (MFI of cells without m-calpain activity minus MFI of cells without both μ- and m-calpain activity) and activity of m-calpain as corrected MFI units obtained by subtraction of 4 from 2 (MFI of cells with all CMAC-tBOC cleaving activity intact minus MFI of cells without m-calpain activity). A representative set of overlayed histograms illustrating the CMAC-tBOC fluorescence of the FMO, substrate-only, substrate + CI-II, and substrate + CI-IV at rest and after 1 hour of stimulation with immobilized anti-CD3 is shown as supplementary figure 1.

### Flow cytometric determination of the influence of calpain inhibition on the activation of phosphorylated p56Lck, PLCγ and NFκB in the resting T cells

Three million PBMC per mLwere treated with either CI II or CI IV, for 0 (untreated control), 10, 20, 30, and 60′ at 37°C, washed, suspended in 0.5 mL of PBS and fixed for 10′ with 4% paraformaldehyde (Biolegend, Burlington, ON). Then the cells were equilibrated by 10′ incubation with PBS containing 10%FBS (Life Technologies). After two washings in PBS, a surface staining (30 minutes on ice in the dark) was performed with anti-CD3-Alexa700, anti-CD4-Phycoerythrin (PE), and anti-CD8-PerCPCy5.5 (prior to staining for pLck505) or with anti-CD3-Alexa700, anti-CD4-Allophycocyanine (APC), and anti-CD8 PerCPCy5.5 (BD Biosciences, Mississauga, ON) prior to staining for other signal molecules. Afterwards the cells were permeabilized with 200 μL of BD Phosflow Perm Buffer III for 30 minutes at 4°C, washed 3 times with ice-cold PBS and stained for 1 hour on ice using anti-pLckY505-Alexa Fluor 647 (BD Biosciences, Mississauga, ON) or one of the primary antibodies: anti-pLck-Y394 (Sigma-Aldrich, Oakville, ON),pPLCγ-Y783 (Abcam, Toronto, ON), and pNFκB-S529 (R&D Systems, Minneapolis, MN), followed by a second intracellular staining (1 hour on ice) with anti-rabbit IgG-PE (Ebioscience, San Diego, CA) prior to flow cytometry data acquisition. The raw parameter used for estimation of the amounts of phosphorylated molecules in untreated and CI-treated T cells was the geometric mean fluorescence intensity (GMFI) from the respective fluorescence channel (PE for anti-pLCK Y394, anti-PLCγ and pNFκB, or APC for anti-pLCK Y505), corrected by subtraction of the background fluorescence (cGMFI) (51). Final parameter defined as %change was calculated as cGMFI(treated) / cGMFI(untreated) * 100 - 100. This way the %change for untreated cells was always equal to 0 and decreased expression of the molecule of interest was shown as negative %change. Statistical significance of the GMFI differences was assessed using the Kolmogorov-Smirnov test [53]. Raw data from a representative experiment are shown as supplementary figure 2.

### T cell stimulation

In order to assess the influence of inhibition of resting calpain activity on the CD4^+^ and CD8^+^ lymphocyte proliferation *in vitro*, PBMC were loaded with BD Horizon™ Violet Cell Proliferation Dye 450 (VPD450™, Becton Dickinson, USA) according to the manufacturer's protocol and cultured for 3 or 5 days with immobilized anti-CD3 antibody (clone OKT3, Ortho Biotech) without or with 10 μMCI II or CI IV. The cultures were performed at 37°C in the RPMI medium supplemented with 10% FBS, 100 U/mLpenicillin and 100 μg/mLstreptomycin (all from Sigma, USA), in a 95% air, 5% CO_2_ humidified incubator. After 72 and 120 hours in culture, the cells, washed, ^+^^+^^+/−^ and fluorescence (including that of VPD450) was recorded by FACS°

### Apoptosis assessment

and possible calpain inhibitor (CI)-induced ^+^^+^*ex vivo*Annexin propidium iodide (PI) staining was applied to distinguish between living, apoptotic and necrotic cellsBecton

### Quantitation of cytokine concentrations in culture supernatants

Concentrations of various cytokines (IFNγ, IL-1β, IL-2, IL-6, IL-8, IL-10, IL-12p70, IL-17 and TNFα) in the culture supernatants of untreated PBMC or of PBMC treated with 10 μMCI-II or CI-IV were determined cytometrically using the Cytokine Beads Array Flex™ kits (Becton Dickinson, USA) according to the manufacturer's instructions. Cytokine concentrations were expressed as picograms (pg) per ml.

### Flow cytometry data acquisition

For the majority of experiments described above it was performed with FACSVerse™ flow cytometer, while acquisition of data concerning the detection of phosphorylated signal molecules (phos-flow) - with FACS Aria III™ cytometer, and with their respective on-board acquisition software (both Becton Dickinson, USA). At least 3*10^4^ cells were acquired from each *ex vivo* sample and at least 5*10^4^ cells were acquired from each sample of PBMC cultured for 72 or 120 hours. Resulting raw data were analyzed using the FlowJo™ and Flowing Software 2.5.1 (© Perttu Terho, Turku University) analysis programs. Analysis of changes in the amounts of phosphoproteins was performed with the help of cloud-based Cytobank™ platform. At analysis, the lymphocytes were first identified by forward and side scatter gating and then by CD3, CD4, and CD8 expression, respectively (with further identification of T cell subpopulation according to the staining scheme used); finally, the signals from channels corresponding to the molecule(s) of interest (CCS, cleaved tBOC, and phosphoproteins) were acquired for the subpopulations defined as above.

### Cytometric separation of CD4^+^ and CD8^+^ lymphocytes

Pure populations of resting CD4^+^ and CD8^+^ lymphocytes were obtained from PBMC stained with appropriate antibody conjugates and sorted using the FACS Aria III™ sorter (Becton Dickinson). At least one million 98% pure CD4^+^ and CD8^+^ lymphocytes had been obtained from each sample. Purified cells were centrifuged, the pellets flash frozen in liquid nitrogen and stored at −80°C until further processing.

### Quantitative real time-PCR estimation of CCS genes' expression

Quantitative Real Time PCR was performed as described elsewhere [12]. Briefly, total RNA was isolated from samples of pure CD4^+^ and CD8^+^ cells using the miRNeasy Mini Kit (Qiagen, Netherlands). DNA removal was carried out using the RNase-Free DNase Set (Qiagen, Netherlands). The quality of the obtained RNA was measured by Agilent 2100 Bioanalyzer using the Agilent RNA 6000 Nano Kit and samples exhibiting high RNA quality were converted to cDNA using the ImProm-II Reverse Transcription System (Promega, USA). Quantitative Real-Time PCR was then performed with the use of the LightCycler FastStart DNA Master SYBR Green I (Roche) and run on the LightCycler 2.0 instrument supplied with the LightCycler Software 4.05 (Roche Diagnostics, Germany). The following sequence of primers (BLIRT, Poland) was selected for Real-Time PCR reactions: 5′-ATTTCGTTTGCTGCCTGGTG-3′ and 5′-ATGGTCAGCTGCAACCACTTA-3′ for μ-calpain, 5′-GCATACGCCAAGATCAACGG-3′ and 5′-GGAGGGGGCTTCTTCAACTC-3′ for m-calpain, 5′-CCCAAGCCTCGGAGTGAATC-3′ and 5′-AGCGGCCTTAGATTCTTCTGT-3′ for calpastatin, and finally 5′-CAAGAAAGTTTGCCTATCTGGGG-3′ and 5′-TCCGGTAGTGGATCTTGGCT-3′ for RPL13A as the reference gene. The reaction consisted of a pre-incubation step (95°C, 10′), a quantification step of 40 cycles (95°C, 10 seconds; 62°C, 10 seconds; and 72°C, 5 seconds per cycle), a melting step (65°C, 15 seconds) and a cooling step (40°C, 30 seconds). The obtained Ct values of μ-calpain, m-calpain, calpastatin and the reference gene RPL13A were analyzed using the ΔΔCt method and expressed as fold change over the expression of RPL13A [54, 55].

### Statistical analyses

Statistical analyses were performed with STATISTICA 8™ (StatSoft, Poland) using the unpaired or paired Student T test or non-parametric Mann-Whitney U-test where applicable. All values are shown in the graphs either as scatter plots with marked means ^+^/− standard deviation (SD), or as bars corresponding to means with marked SD

## SUPPLEMENTARY MATERIALS FIGURES


