# Supplementary material for: Roles of calpain-calpastatin system (CCS) in human T cell activation
Source: Oncotarget. 2016 Nov 9;7(47):76479–95. doi: 10.18632/oncotarget.13259 (PMC5363525; doi:10.18632/oncotarget.13259)
Supplement: Supplementary file 1 [file oncotarget-07-76479-s001.pdf]

Roles of calpain-calpastatin system (CCS) in human T cell activation

Supplementary Material

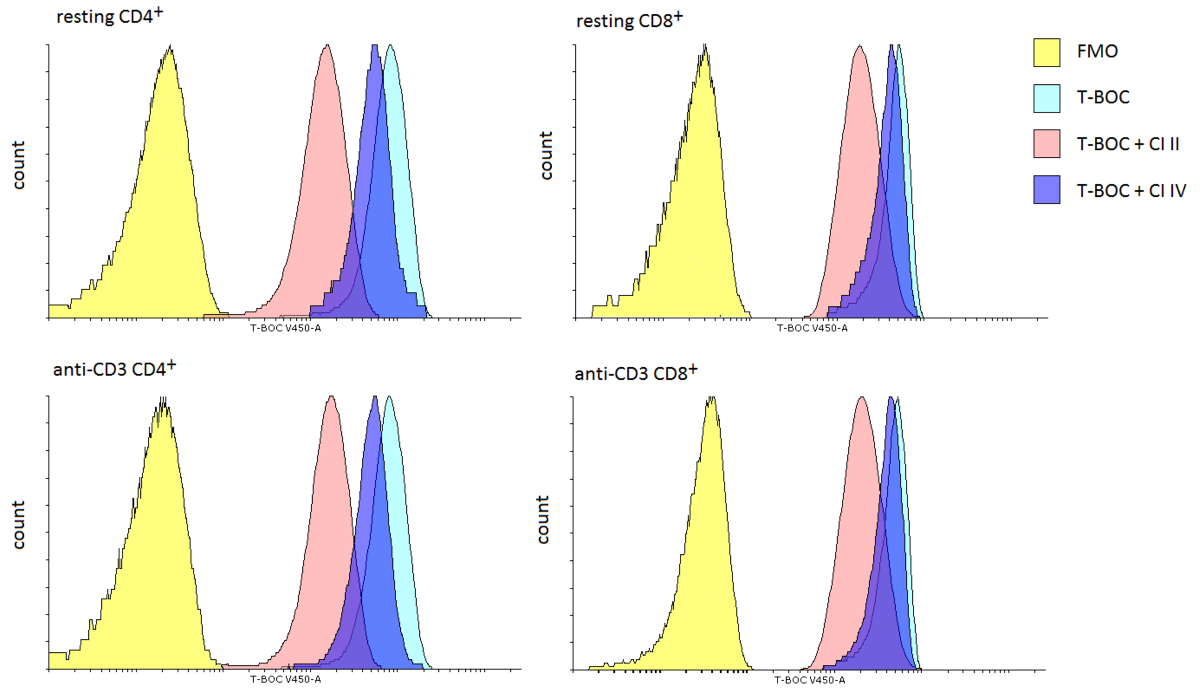

Supplementary Fig. 1. Results of a representative experiment showing the activity of calpains in PBMC-derived CD4+ lymphocytes ex vivo (resting) and after 1 h stimulation with immobilized anti-CD3. Representative results of cytometric determination of relative activities of μ- and m-calpains in resting (upper panels) and anti-CD3-stimulated (lower panels) $CD4^+$ and $CD8^+$ lymphocytes. PBMC were loaded with CMAC-tBOC without or after preincubation with either calpain inhibitor II (CI-II) or calpain inhibitor IV (CI-IV) as described in Materials and Methods. Fluorescence minus one (FMO) from the channel recording the CMAC-tBOC fluorescence with cells surface-stained for phenotype, but without the calpain substrate has been recorded and show as negative control. Observe that: CMAC-tBOC fluorescence distribution is Gaussian (indicating that all $CD4^+$ and $CD8^+$ lymphocytes exhibit the enzyme activity both at rest and after stimulation, and allowing for the use of mean fluorescence intensity (MFI) as the measure); inhibition by CI-II is stronger than that exerted by CI-IV.

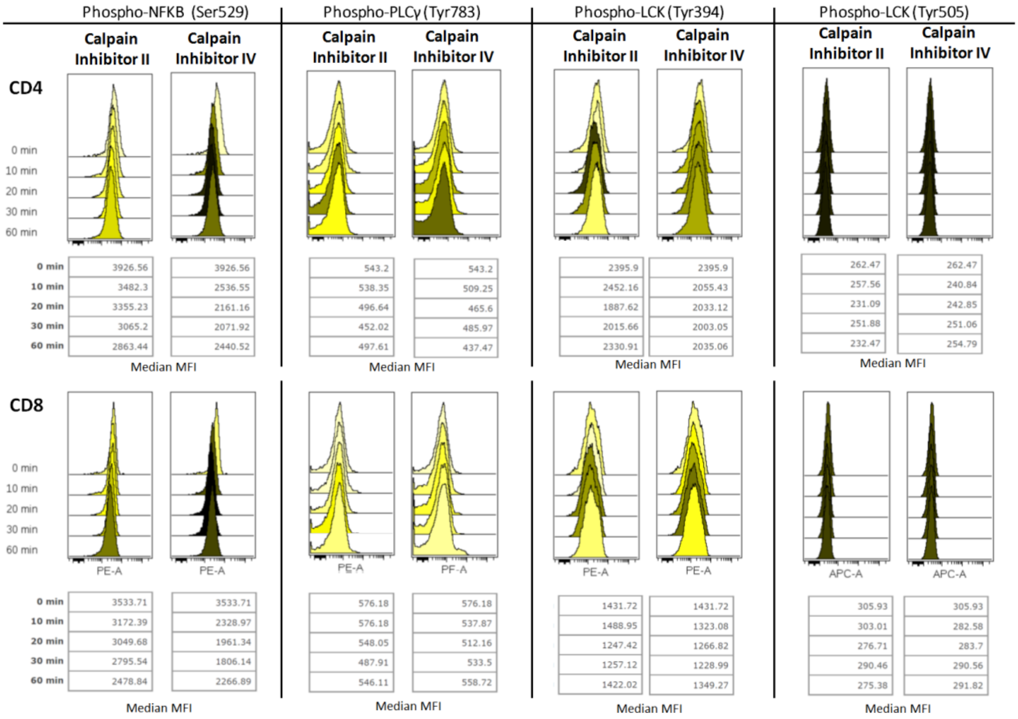

Supplementary Fig. 2. Results of a representative experiment showing the reduction of the amount of chosen phospho-proteins related to calpain inhibition in resting T cells. Representative results of determination of in vitro influence of calpain inhibitors II and IV on the amounts of phosphorylated NFκB (Phospho-NFKB), PLCγ (Phospho-PLCγ), and p56Lck (Phospho-LCK) in human resting peripheral blood $CD4^+$ and $CD8^+$ lymphocytes. Displayed histograms were obtained at times 0 min, 10 min, 20 min, 30 min and 60 min from the start of incubation with respective calpain inhibitor. Median fluorescence (Median MFI) values indicating the strength of phosphor-protein - associated signals are shown below each set of histograms. The experiments were performed as in Materials and Methods. The analysis was done using the cloud-based Cytobank™ software. The experiments were performed in triplicate and gave similar results.
